# Activation of apoptosis in NAF-1-deficient human epithelial breast cancer cells

**DOI:** 10.1242/jcs.178293

**Published:** 2016-01-01

**Authors:** Sarah H. Holt, Merav Darash-Yahana, Yang Sung Sohn, Luhua Song, Ola Karmi, Sagi Tamir, Dorit Michaeli, Yuting Luo, Mark L. Paddock, Patricia A. Jennings, José N. Onuchic, Rajeev K. Azad, Eli Pikarsky, Ioav Z. Cabantchik, Rachel Nechushtai, Ron Mittler

**Affiliations:** 1 Department of Biological Sciences, University of North Texas, Denton, TX 76203, USA; 2 The Alexander Silberman Institute of Life Science, Hebrew University of Jerusalem, Edmond J. Safra Campus at Givat Ram, Jerusalem 91904, Israel; 3 Department of Chemistry & Biochemistry, University of California at San Diego, La Jolla, CA 92093, USA; 4 Center for Theoretical Biological Physics andDepartment of Physics, 239 Brockman Hall, 6100 Main Street-MS-61, Rice University, Houston, TX 77005, USA; 5 Department of Mathematics, University of North Texas, Denton, TX 76203, USA; 6 Department of Immunology and Cancer Research, Institute for Medical Research Israel Canada (IMRIC), Hebrew University-Hadassah Medical School, Jerusalem 91120, Israel

**Keywords:** NEET proteins, NAF-1, Mitochondria, ROS, Apoptosis, Cancer

## Abstract

Maintaining iron (Fe) ion and reactive oxygen species homeostasis is essential for cellular function, mitochondrial integrity and the regulation of cell death pathways, and is recognized as a key process underlying the molecular basis of aging and various diseases, such as diabetes, neurodegenerative diseases and cancer. Nutrient-deprivation autophagy factor 1 (NAF-1; also known as CISD2) belongs to a newly discovered class of Fe-sulfur proteins that are localized to the outer mitochondrial membrane and the endoplasmic reticulum. It has been implicated in regulating homeostasis of Fe ions, as well as the activation of autophagy through interaction with BCL-2. Here we show that small hairpin (sh)RNA-mediated suppression of NAF-1 results in the activation of apoptosis in epithelial breast cancer cells and xenograft tumors. Suppression of NAF-1 resulted in increased uptake of Fe ions into cells, a metabolic shift that rendered cells more susceptible to a glycolysis inhibitor, and the activation of cellular stress pathways that are associated with HIF1α. Our studies suggest that NAF-1 is a major player in the metabolic regulation of breast cancer cells through its effects on cellular Fe ion distribution, mitochondrial metabolism and the induction of apoptosis.

## Abstract (precis)

**Summary:** NAF-1 is a major player in the metabolic regulation of breast cancer cells through its effects on cellular Fe ion distribution, mitochondrial metabolism and the induction of apoptosis.

## INTRODUCTION

Iron (Fe) ions (which can exist in multiple oxidations states, most commonly Fe^2+^ and Fe^3+^) are essential for many cellular processes, including energy metabolism, DNA synthesis and cell cycle progression (Crichton, 2009). An important physiological contribution of Fe ions is associated with the formation of iron-sulfur (Fe-S) clusters (Lill, 2009), a process that takes place initially and predominantly in mitochondria but comprises obligatory cytosolic steps (Lill et al., 2014; Maio and Rouault, 2015; Stehling et al., 2014). Because Fe ions are linked to many essential processes in the cell, as well as to the formation of reactive oxygen species (ROS) through the Fenton reaction, a disruption in cellular Fe ion distribution can have major effects on cellular metabolism, potentially leading to oxidative stress and activation of cell death pathways (Halliwell and Gutteridge, 2007). As cancer cells are voracious consumers of Fe ions, treatments that disrupt their Fe ion balance (Torti and Torti, 2013) and/or affect their redox status (Bystrom et al., 2014; Bystrom and Rivella, 2015) have been investigated as potential therapeutic targets.

NEET proteins are a novel class of Fe-sulfur (2Fe-2S) cluster-containing proteins localized to the outer mitochondrial, endoplasmic reticulum (ER) and mitochondria-associated membranes (MAM) membranes, and are defined by a unique CDGSH amino acid sequence at their Fe-S-cluster-binding domain (Tamir et al., 2015). Mitochondrial (mito)NEET (mNT; encoded by *CISD1*) and NAF-1 (encoded by *CISD2*) are the most studied representatives of the three-member NEET-family in humans. They have been implicated in a number of pathologies, including neural development, obesity, diabetes and aging, and are rapidly gaining prominence as targets for cancer therapy (Bai et al., 2015; Boucquey et al., 2006; Chen et al., 2009b,c, 2010; Liu et al., 2014; Tamir et al., 2015; Wang et al., 2014a,b; Wiley et al., 2013; Wu et al., 2012). NAF-1 is located at the interface of the ER and mitochondria, anchored to the Ca^2+^ channel inositol 1,4,5-triphosphate receptor and is necessary for BCL-2-mediated suppression of autophagy and control of Ca^2+^ homeostasis (Chang et al., 2012a,b, 2010; Du et al., 2015). In the absence of NAF-1, the autophagy-promoting Beclin1 complex dissociates from BCL-2, and autophagy is activated (Sohn et al., 2013; Tamir et al., 2015). Although the binding of NAF-1 to BCL-2 has been mapped in a recent study (Tamir et al., 2014), the potential of NAF-1 to activate apoptosis is unclear.

Here, we report that small hairpin (sh)RNA-mediated suppression of NAF-1 in human breast cancer cells results in the activation of apoptosis in xenograft MDA-MB-231 tumors and in MCF-7 or MDA-MB-231 cells grown in culture. Suppression of NAF-1 expression resulted in increased uptake of Fe ions into cells that was followed by an accumulation of Fe ions in mitochondria and enhanced mitochondrial ROS production. Metabolomics and transcriptomics analysis of breast cancer cells in which NAF-1 had been suppressed revealed a further shift towards glycolysis and glutaminolysis, and the activation of cellular stress pathways associated with HIF1α. Suppression of NAF-1 expression in human breast cancer cells appears, therefore, to reduce their tumorigenicity by interfering with cellular Fe ion distribution and energy metabolism, resulting in the enhanced accumulation of Fe ions and ROS in the mitochondria, and the activation of apoptosis.

## RESULTS

### Tumor cells with suppressed levels of NAF-1 contain damaged mitochondria, and show signs of autophagy and apoptosis activation

**Fig. 1.**
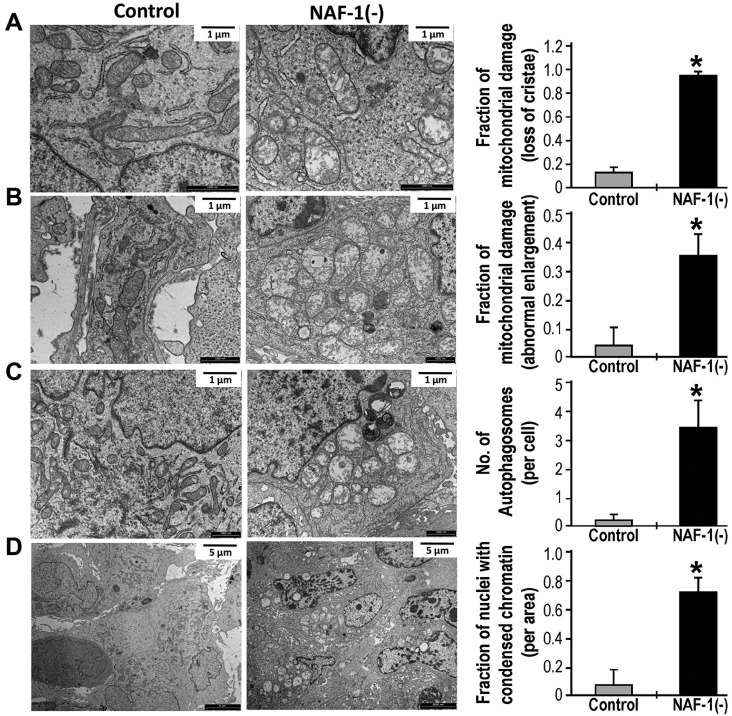
**Mitochondrial damage and activation of autophagy/apoptosis in xenograft tumors derived from control or NAF-1(−) MDA-MB-231 cells.** Representative transmission electron microscopy (TEM) images of tumors from mice that had been injected with control MDA-MB-231 cells (left) and mice that had been injected with NAF-1(−) MDA-MB-231 cells (middle) are shown side-by-side, and graphs (right) show quantitative analysis of mitochondrial damage in the form of loss of crista (A) and abnormal elongation (B), the accumulation of autophagosomes (C) and the number of nuclei with condensed chromatin (D). *Significant difference at *P*<0.05 (*n*=20 different sections; *t*-test). Error bars represent mean±s.d.

To gain insight into the function of NAF-1 in tumor growth, we conducted transmission electron microscopy (TEM) studies of control and shRNA-suppressed xenograft tumors grown, as described in Sohn et al., 2013. As shown in Fig. 1, mitochondria in the NAF-1-depleted [NAF-1(−)] tumors exhibited a significant loss of cristae, with the remaining cristae disorganized and distorted in structure. Suppression of NAF-1 also resulted in a significant increase in the number of enlarged mitochondria with a swollen, rounded phenotype (Fig. 1). Suppression of NAF-1 in MDA-MB-231 cells grown in tumors resulted in an increased number of autophagosomes, evidence of increased autophagy (Fig. 1). Moreover, tumors in which NAF-1 had been suppressed contained a significantly higher percentage of cells with condensed chromatin, a hallmark of apoptosis (Fig. 1).

### Tumors derived from MDA-MB-231 cells with suppressed NAF-1 expression contain a significantly higher number of cells with activated caspase-3 and elevated γH2AX

**Fig. 2.**
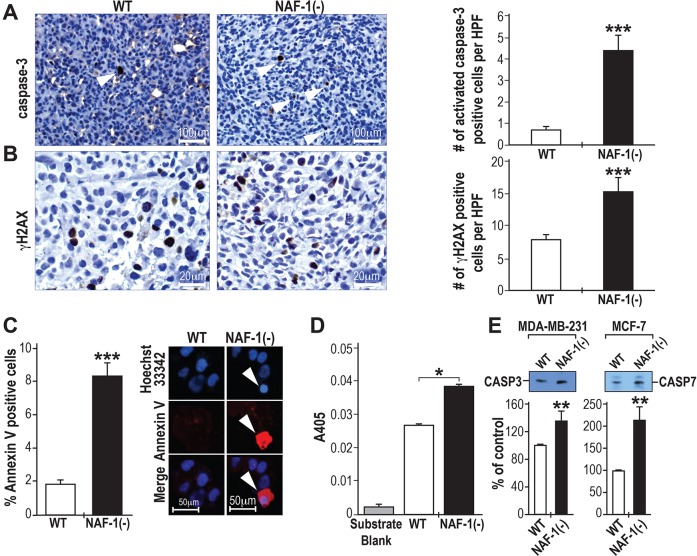
**Activation of apoptosis in xenograft tumors and cancer cells with suppressed levels of NAF-1.** (A) Left, immunohistochemistry (IHC) analysis using an antibody against activated caspase-3 showing a higher number of active-caspase-3-positive cells in NAF-1(−) tumors. Active-caspase-3-positive cells are marked by white arrowheads. Right, quantification of staining of active caspase-3. (B) Left, IHC analysis using an antibody against γH2AX showing a higher number of γH2AX-positive cells in NAF-1(−) tumors. Right, quantification of γH2AX staining. For A,B, cells were counted in ten high-power fields (×40) for each section obtained from five mice in each group; ***P<0.001 (*t*-test). (C) Left, activation of apoptosis in NAF-1(−) MDA-MB-231 cells observed by using annexin-V staining; ***P<0.001 (*t*-test). Right, representative apoptotic NAF-1(−) cells are indicated by the white arrowhead. Nuclei are counterstained with Hoechst 33342. (D) NAF-1(−) MDA-MB-231 cells show increased caspase-3 enzymatic activity, as measured by using a colormetric activity assay; *P<0.05 (*t*-test). (E) Western blot analysis showing the accumulation of activated caspase-3 and caspase-7 in MDA-MB-231 and MCF-7 cells in which NAF-1 expression had been suppressed. Protein expression was calculated as a percentage of that of control from three different experiments. **P<0.01 (*n*=3; *t*-test). Error bars represent mean±s.d. WT, wild type.

To determine if apoptosis is activated in tumors in which NAF-1 expression had been suppressed, we conducted immunohistochemical analysis on NAF-1(−) tumors using an antibody against activated caspase-3. As shown in Fig. 2A, the number of cells containing activated caspase-3 was higher in tumors derived from NAF-1(−) cells compared to those derived from negative controls. As shown in Fig. 2B, the number of tumor cells containing elevated levels of γH2AX, a marker for the activity of the DNA damage response pathway, was also high in tumors derived from NAF-1(−) cells compared to tumors derived from control cells.

### Activation of apoptosis in MCF-7 and MDA-MB-231 cells with suppressed NAF-1 expression in culture

Additional support for the activation of apoptosis in NAF-1(−) cells was obtained by using annexin-V staining (annexin-V conjugated to Alexa-Fluor-555). As shown in Fig. 2C, compared to control cells, a significant increase in the number of apoptotic cells was observed in NAF-1(−) MDA-MB-231 cells that had been grown in culture. In addition, as shown in Fig. 2D, cell extracts obtained from NAF-1(−) MDA-MB-231 cells that had been grown in culture had a higher level of caspase-3 enzymatic activity compared to cell extracts obtained from control cells. The protein level of activated caspase-3 and caspase-7 was also higher in NAF-1(−) MDA-MB-231 and NAF-1(−) MCF-7 cells, respectively, as determined by western blotting (Fig. 2E; caspase-3 function in MCF-7 cells is mediated through caspase-7).

### Suppression of NAF-1 in MDA-MB-231 and MCF-7 cells resulted in increased uptake of Fe ions into cells and mitochondria, as well as enhanced mitochondrial ROS production

**Fig. 3.**
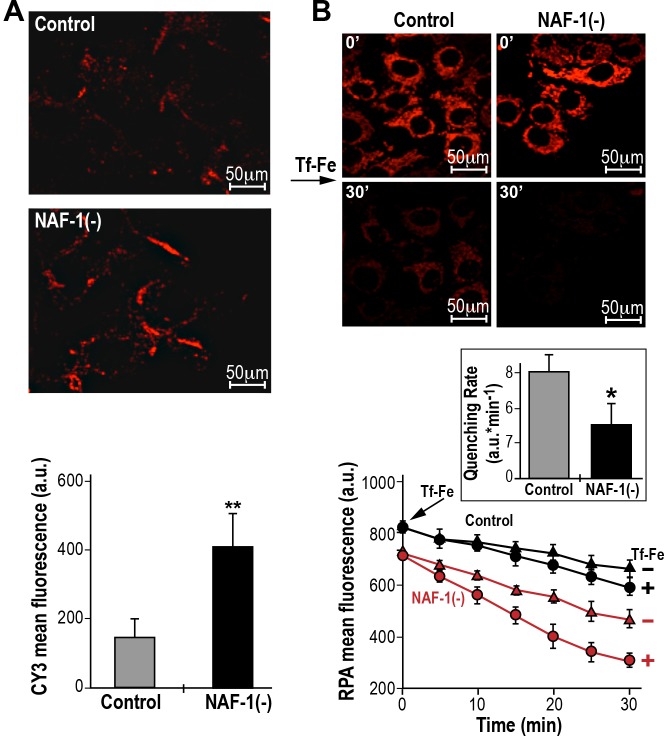
**Elevated levels of TfR and Fe ion uptake into mitochondria in NAF-1(−) MCF-7 cells.** (A) Elevated levels of the TfR protein on the membranes of NAF-1(−) cells. Top, semi-confocal microscopy images of NAF-1(−) cells and control cells. Cells were immunostained with an antibody against transferrin and counterstained with Cy3 (red). Bottom, quantitative representation of mean fluorescence intensity (5 cells/field; *n*=3 independent experiments); **P<0.01 (*t*-test). (B) Elevated Fe ion uptake into mitochondria of NAF-1(−) cells. Mitochondria were loaded with red fluorescent RPA (which undergoes quenching upon binding of Fe ions), exposed to transferrin-bound Fe ions (Tf-Fe; 2 µM) and imaged over time. Top, semi-confocal microscopy images of NAF-1(−) and control cells control cells following 30 min of incubation with transferrin-bound Fe ions. Middle, bar graph showing the corrected slopes for net quenching associated with administration of transferrin-bound Fe ions [fluorescence (a.u.)×min^−1^±s.e.); *P<0.05 (*t*-test). Bottom, line graphs showing the rates of quenching [iron ingress; fluorescence (a.u.)×min^−1^], calculated for *n*=3 independent experiments. Error bars represent mean ±s.d.

**Fig. 4.**
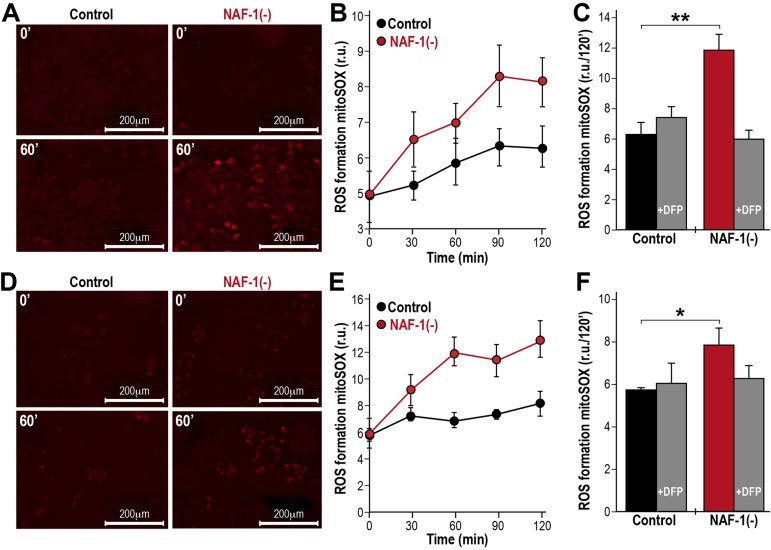
**Enhanced mitochondrial ROS production in NAF-1(−) cells.** (A) Images of mitoSOX-loaded control and NAF-1(−) MDA-MB-231 cells at 0 and 60 min of incubation. (B) Line graph showing a time-course of the change in mitoSOX fluorescence in mitoSOX-loaded control and NAF-1(−) MDA-MB-231 cells. (C) Bar graph showing the suppression of mitochondrial ROS production in mitoSOX-loaded control and NAF-1(−) MDA-MB-231 cells at 120 min of incubation by pretreatment with DFP (100 μM). (D–F) As A–C but using MCF-7 cells. *n*=3 independent experiments; *P<0.05, **P<0.01 (*t*-test). r.u., relative units. Error bars represent mean±s.d.

To explore the potential of NAF-1 to affect Fe ion and ROS metabolism in cancer cells (Tamir et al., 2015), we examined the level of expression of transferrin receptors and the site of ROS accumulation in breast cancer cells with suppressed NAF-1 expression. As shown in Fig. 3A and Fig. S1A, suppression of NAF-1 expression in MCF-7 (Fig. 3A) or MDA-MB-231 cells (Fig. S1A) resulted in increased expression of the transferrin receptor protein (TfR; also known as TFRC) at the plasma membrane. The increased expression of TfR at the plasma membrane of cells in which NAF-1 had been suppressed was accompanied by an increased uptake of transferrin-bound Fe into cells, which we assessed by tracing the ingress of Fe into mitochondria with the fluorescent probe Rhodamine-B [(1,10-phenanthrolin-5-yl)aminocarbonyl]benzyl ester (RPA) serving both as mitochondrial Fe-ion sensor and as an Fe-ion trap (Fig. 3B, Fig. S1B; Breuer et al., 2008; Sohn et al., 2013; Cabantchik et al., 2014). These results indicated that NAF-1 suppression resulted in an increased uptake of Fe ions into cells. However, to what extent the mitochondria-accumulated Fe ions were in labile forms demanded the examination of its catalytic involvement in ROS production. As shown in Fig. 4 and Fig. S2, enhanced ROS production was measured by using mitoSOX Red (Fig. 4) or dihydroethidium (DHE; Fig. S2) and observed in NAF-1(−) cells. Moreover, as also shown in Fig. 4 and Fig. S2, ROS accumulation in NAF-1(−) cells was largely prevented through pre-treatment of cells with the Fe-ion chelator deferiprone (DFP). The latter provides a direct link between Fe ion accumulation and ROS production, pointing towards mitochondria as the linkage milieu (Fig. 4).

The alterations in Fe ion and ROS metabolism caused by NAF-1 suppression (Figs 3, 4, Figs S1, S2) could have a dramatic effect on many different pathways in cancer cells, causing the activation of apoptosis and the suppression of tumor growth. To further dissect the molecular and cellular consequences of NAF-1 suppression, we conducted a detailed metabolomics and transcriptomic analysis of MCF-7 cells in which NAF-1 expression had been suppressed, focusing on energy metabolism and the activation of cell survival and cell death pathways.

### Alterations in energy metabolism in cancer cells with suppressed NAF-1 expression

**Fig. 5.**
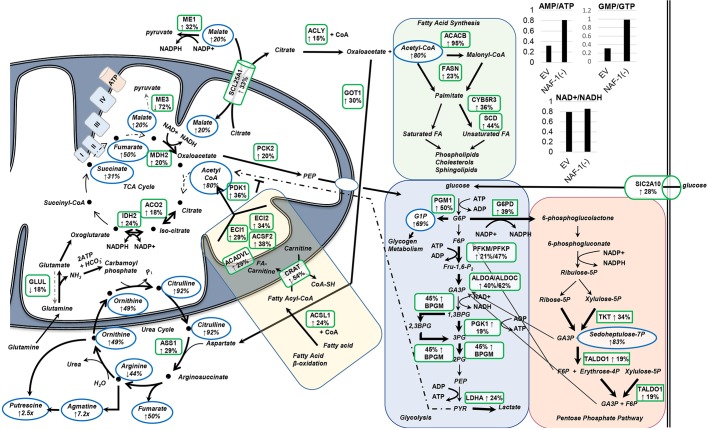
**Alterations in the levels of metabolites and mRNA transcripts that are crucial to energy metabolism in NAF-1(−) MCF-7 cells.** Metabolic pathway maps showing the metabolites and transcripts that are substantially altered in NAF-1(−) MCF-7 cells and that are involved in glycolysis, the pentose phosphate pathway, fatty acid synthesis and β-oxidation, the TCA cycle and the urea cycle. Altered metabolites are outlined with blue ovals with the alteration indicated with arrows, and the percentage or fold change is given below each. Genes whose transcription is altered are outlined with a green box with the alteration indicated by arrows, and the percentage or fold change is given below each. Only metabolites and transcripts that showed a significant change (*P<0.05) in NAF-1(−) compared to negative controls are shown. Reactions that are potentially increased due to increases of metabolite or transcription are indicated by heavier arrow weights. Reactions that are potentially decreased, as indicated by decreases in metabolites or transcription are indicated by lighter, dashed arrow weights. Results indicate increased glycolysis and fatty acid metabolism, with possible dysfunction of the TCA cycle requiring utilization of glutamine (with possible glutamine-dependent reductive carboxylation) and consequently increased flux through the urea cycle. The inset in the figure showing graphs is a comparison of the AMP:ATP, GMP:GTP and NAD+:NADH ratios between MCF7 NAF-1(−) and MCF7 scrambled-vector controls.

Metabolomics and RNA-Seq analysis were performed on NAF-1(−) and scrambled-vector control MCF-7 cells that had been grown in culture. Out of the 386 metabolites detected, 132 were found to be significantly altered (*P*<0.05, ANOVA; Table S1). RNA-Seq analysis identified 1585 significantly differentially expressed transcripts (*q*<0.05), with 756 significantly increased and 828 significantly decreased (Tables S2 and S3, respectively). Dissecting this data with respect to energy metabolism, we found that NAF-1(−) cells had significantly decreased levels of ATP and GTP, with a corresponding increase in AMP, suggestive of less available energy and/or a possible decline in mitochondrial function (Fig. 5; Table S1).

Further investigation revealed significantly increased accumulation of metabolites through the urea cycle and downstream polyamine biosynthesis in NAF-1(−) cells compared to negative control (Fig. 5), indicating increased glutamine metabolism (Ko et al., 2011; Meng et al., 2010). The accumulation of tricarboxylic acid (TCA) cycle intermediates – succinate, fumarate and malate – in NAF-1(−) cells suggested defects in flow throughout the electron transport chain (Fig. 5), and could reflect an association between complex II – also called succinate dehydrogenase, which is dependent on Fe-S clusters for proper assembly and function – and Fe ion regulation that is affected by NAF-1 deficiency (Fig. 3; Fig. S1). Malate and fumarate can both also be formed in reactions outside of the TCA cycle – malate as a part of the malate–aspartate shuttle and fumarate as a by-product of argininosuccinate catabolism in the urea cycle. Increases in these metabolites in NAF-1(−) cells could indicate redirection of these pathways to cope with the loss of mitochondrial function that is caused by suppression of NAF-1, as well as an attempt to sustain synthesis of necessary biosynthetic intermediates for proliferation. The increased glutamine metabolism combined with deficiencies in the electron transport chain (Sohn et al., 2013) could indicate that NAF-1(−) cells utilize reductive carboxylation, converting glutamine-derived oxoglutarate to citrate, reversing the isocitrate dehydrogenase reaction to maintain production of TCA cycle intermediates necessary for cell growth (Fig. 5; Mullen et al., 2014, 2012).

RNA-Seq analysis revealed significantly increased transcript levels of key genes that are involved in glycolysis and the pentose phosphate pathway (Fig. 5). This finding suggests that the loss of NAF-1 caused an alteration in the transcriptional program of cells, shifting them further away from a mitochondrial energy program, to become more dependent on cytosolic ATP production for survival.

### Enhanced susceptibility of NAF-1(−) cancer cells to a glycolytic inhibitor

**Fig. 6.**
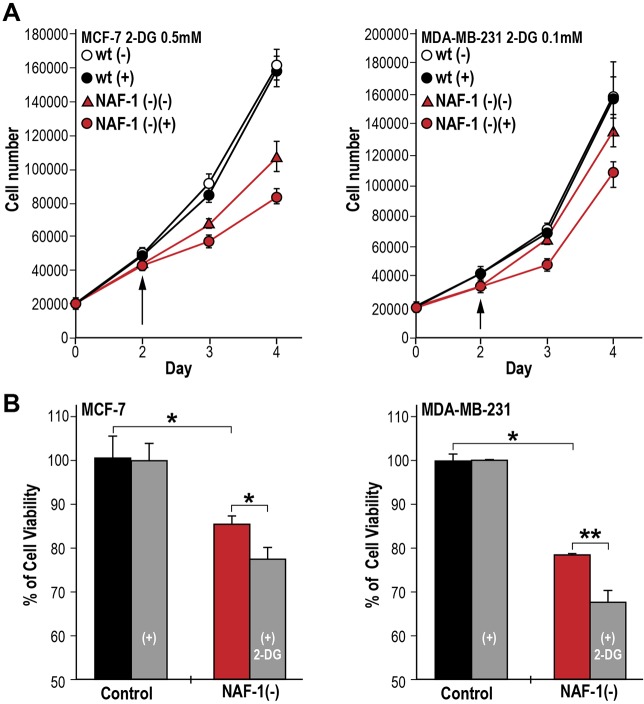
**The effect of 2-DG on cell growth and viability of control and NAF-1(−) MCF-7 and MDA-MB-231 cells.** (A) Suppression of cell growth in NAF-1(−) MCF-7 (left) and MDA-MB-231 (right) cells that had been treated with 0.5 or 0.1 mM 2-DG, respectively, compared to control cells. Arrows indicate the point of 2-DG addition to the cultures. (B) Alamar-Blue cell viability measurements of NAF-1(−) MCF-7 (left) and MDA-MB-231 (right) cells at days 4 and 5 following the addition of 0.4 and 0.1 mM 2-DG, respectively. Results indicate that 2-DG at the concentrations used (0.5 and 0.1 mM) had no significant effect on the growth or viability of control (WT), whereas it had a significant effect on the growth and cell viability of NAF-1-suppressed MCF-7 and MDA-MB-231 cells. Concentrations higher than 2 mM caused complete growth inhibition of WT and NAF-1(−) cells (data not shown). Mean±s.d. from three individual experiments; *P<0.05, **P<0.01 (*t*-test).

To test whether the changes in metabolite and transcript levels identified in NAF-1(−) cells (Fig. 5) indeed resulted in a further shift of NAF-1(−) cells toward dependency on glycolysis compared to control breast cancer cells, we grew MCF-7 and MDA-MB-231 control and NAF-1(−) cells in the presence or absence of the glycolysis inhibitor 2-deoxy-d-glucose (2-DG). At high concentrations of 2-DG, both control and NAF-1-suppressed MCF-7 and MDA-MB-231 cells died (data not shown). However, as shown in Fig. 6, we were able to identify specific concentrations of 2-DG that resulted in differential effects on the growth and viability of NAF-1(−) MCF-7 or MDA-MB-231 cells compared to their corresponding controls. The growth (Fig. 6A) and viability (Fig. 6B) of MCF-7 or MDA-MB-231 cells with suppressed expression of NAF-1 was therefore significantly reduced in the presence of 2-DG, compared to their corresponding controls. These findings support our metabolomic and transcriptomic analyses, demonstrating that suppression of NAF-1 results in a metabolic shift of cancer cells to be even more dependent on glycolysis, making NAF-1(−) cells more susceptible to the glycolysis inhibitor 2-DG compared to control cells.

### Activation of cellular stress and survival pathways in cancer cells in which NAF-1 expression has been suppressed

**Fig. 7.**
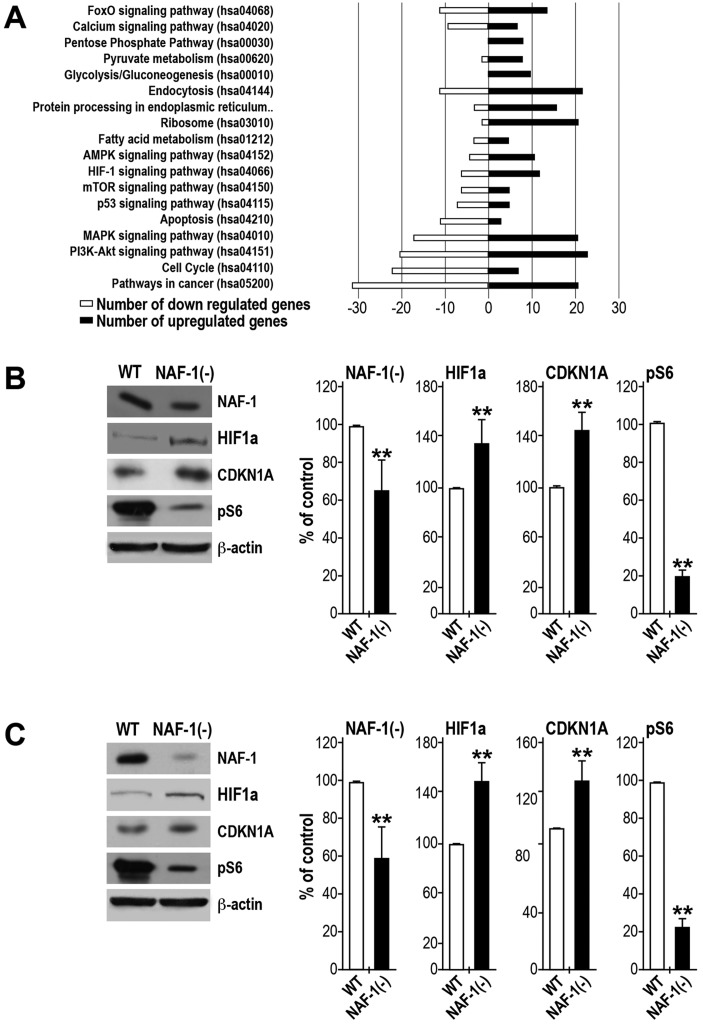
**Activation of stress and cellular survival pathways in cancer cells in which NAF-1 express has been suppressed.** (A) Differentially expressed genes in NAF-1(−) MCF-7 cells relative to those in MCF7 negative controls that are found in KEGG human-specific signaling pathways. Black bars represent the number of genes of which the expression is increased in each pathway, and white bars represent the number of genes of which the expression is decreased in each pathway. (B,C) Western blot analysis and quantification (bar graphs) of wild-type (WT) and NAF-1(−) MCF-7 (B) or MDA-MD-231 (C) cells showing the protein levels of NAF-1, HIF1α, CDKN1A and pS6. β-actin was used as a loading control. Protein expression was calculated as a percentage of that of control from three different experiments. **P<0.01 (*t*-test), *n*=3.

Dissecting the RNA-Seq data by comparing NAF-1(−) MCF-7 cells with scrambled-vector controls revealed significantly increased transcript levels of a set of stress-response transcripts (Fig. 7A; Table S4). Those classified by using the KEGG Pathway database (Figs 5 and 7A) were heavily represented in glucose metabolism pathways, including glycolysis, the pentose phosphate pathway and pyruvate metabolism, as well as in many stress-related pathways, including AMPK, HIF1α, mammalian target of Rapamycin (mTOR) and tumor protein p53 (also known as TP53) signaling.

Significantly upregulated stress and DNA-damage-related transcripts included known gene targets of the transcription factors HIF1α and/or p53, such as GDF15, BNIP3, CDKN1A (p21), NDRG1, GADD45A, GADD45G, MIF, DDIT4, STK11 (LKB1) and OSGIN1 (Table S4). These pathways might have been activated through two routes – stabilization of HIF1α due to altered Fe ion metabolism and/or increased ROS accumulation, or ROS-induced DNA damage leading to stabilization of p53 (Chepelev and Willmore, 2011; Mole, 2010; Reinhardt and Schumacher, 2012). The upregulation of CDKN1A, GADD45A, GADD45G and DDIT4 suggests that NAF-1(−) cells could undergo cell cycle arrest (Table S4) (Abbas and Dutta, 2009; Salvador et al., 2013; Sofer et al., 2005). In addition, the increased expression of BNIP3, DDIT4 and OSGIN1 might support the notion of activation of apoptosis (Table S4) (Burton and Gibson, 2009; Hu et al., 2012; Mellor and Harris, 2007; Yao et al., 2008). NAF-1(−) cells were also found to display significantly increased levels of known Nrf2 (also known as NFE2L2) stress-related target genes, including HMOX1, BLVRB, FTH1 and NQO1 (Ishii et al., 2000; Itoh et al., 1997; Mitsuishi et al., 2012; Wu et al., 2011). Nrf2 is stabilized through multiple routes (Kansanen et al., 2013). In NAF-1(−) cells, Nrf2 could be stabilized due to elevated ROS levels and/or the increased expression of HIF1α- and p53-target protein p21 (CDKN1A; Table S4) (Chen et al., 2009a; Chepelev and Willmore, 2011; Villeneuve et al., 2009). NAF-1(−) cells displayed significantly elevated transcript levels of the glycolysis and pentose phosphate transition genes ALDOA, ALDOC, G6PD, TALDO1, TKT, PGK1 and LDHA (Table S4; Mitsuishi et al., 2012; Wu et al., 2011). Additionally, NAF-1(−) cells have significantly increased expression of pyruvate dehydrogenase kinase 1 (PDK1), which inactivates the mitochondrial multi-enzyme pyruvate dehydrogenase complex through phosphorylation in response to hypoxia and/or oxidative stress (Kim et al., 2006). This could ensure that pyruvate does not enter the TCA cycle and function to downregulate mitochondrial energy production.

The changes observed in the transcriptome and metabolome of NAF-1(−) cells could reflect the activation of different stress-response pathways associated with AMPK, mTOR, p53 and/or HIF1α (Figs 5 and 7A). To initiate a more detailed study into the response of cancer cells to NAF-1 suppression, we used western blotting to study the levels of selected proteins involved in these pathways in MCF-7 and MDA-MB-231 cells. As shown in Fig. 7B, NAF-1 suppression resulted in a significant stabilization of HIF1α and in an increase in the level of CDKN1A (p21). In contrast, the level of BNIP3, which should have also been increased as an outcome of HIF1α stabilization, was decreased (Fig. S3). Interestingly, treatment of NAF-1(−) cells with the AMPK inhibitor compound C (dorsomorphin) resulted in increased BNIP3 protein levels (Fig. S3), indicating an activation of AMPK in NAF-1(−) cells (Park et al., 2013). This is in agreement with the increased AMP:ATP ratio observed using metabolomics analysis of NAF-1(−) (Fig. 5) and is supported by the significantly increased expression of the serine/threonine protein kinase STK11 (LKB1) that is known to activate AMPK (Shaw et al., 2004). The possible activation of AMPK led us to investigate the mTOR phosphorylation state of the mTOR target proteins ribosomal protein S6 (pS6, also known as RPS6) and the translational repressor protein 4E-BP1 (Fonseca et al., 2014; Gingras et al., 2001). As shown in Fig. 7B, the mTOR target pS6 was significantly less-phosphorylated in MCF-7 and MDA-MB-231 NAF-1(−) cells, suggesting that loss of NAF-1 resulted in the inactivation of mTOR. The analyses presented in Fig. 7B and Fig. S3 support our transcriptomics and metabolomics analyses and provides an entry point into further proteomics studies of cells with suppressed expression of NAF-1.

## DISCUSSION

Previous studies have suggested that NAF-1 could be involved in maintaining Fe ion homeostasis in cells (Sohn et al., 2013; Tamir et al., 2015). Nevertheless, the precise role of NAF-1 in this process has remained unclear. Here, we show that NAF-1 deficiency results in an increased expression of TfR at the plasma membrane, an increased uptake of transferrin-bound Fe into cells and an increased production of ROS in the mitochondria (Figs 3, 4; Figs S1, S2). Taken together, the alterations in Fe ions and ROS in cancer cells with suppressed NAF-1 expression appear to resemble the alterations in Fe ions and ROS that are induced by the disruption of Fe-S biogenesis in conditions such as Friedreich's ataxia and in some forms of sideroblastic anemia (Napier et al., 2005; Wilson, 2006). NAF-1 could therefore be required for proper Fe-S biogenesis or mobilization in cancer cells. In support of such a possibility are the unique properties of the NAF-1-containing Fe-S cluster, namely that it is labile and could be donated to an apo-acceptor protein, and that it localizes at the interface between the ER, cytosol and mitochondria (Tamir et al., 2013; Wiley et al., 2013). The finding that DFP, a membrane-permeant Fe-ion chelator, inhibits the rise in mitochondrial labile Fe ions (Sohn et al., 2013) and mitochondrial ROS production in NAF-1(−) cells (Fig. 4) suggests that the accumulation of Fe ions in the mitochondria is a primary cause of the enhanced production of mitochondrial ROS in these cells. Although a role for NEET proteins in Fe-S biogenesis has been previously proposed (Ferecatu et al., 2014), and although it is tempting to speculate that NAF-1 deficiency is directly responsible for the changes in Fe ion and ROS homeostasis in cancer cells through this proposed role (that our results, described above, support), further studies are required to support this notion.

The activation of apoptosis in cells with suppressed NAF-1 expression (Figs 1 and 2) could result from the activation of cell death pathways through ROS, mTOR inactivation or HIF1α stabilization (Fig. 7), as well as or as a result from altered interactions of BCL-2 in the absence of NAF-1. Because NAF-1 binds to both the pro- and anti-apoptotic regions (BH3 and BH4) of BCL-2 (Tamir et al., 2014), a decrease in the cellular levels of NAF-1 in cancer cells could reduce its binding to these BCL-2 regions, making them available for interactions with different pro-apoptotic proteins, such as PUMA, NOXA and BAD, which would trigger apoptosis. This possibility requires further studies because it could open the way for the development of new drugs that target the NAF-1–BCL-2 interaction. An example for such a drug could be the cluvenone derivative MAD-28, which binds to NAF-1 within the vicinity of the BCL-2–NAF-1 interaction site (Bai et al., 2015). This small molecule has recently been found to target breast cancer cells without any apparent effect on normal breast cells, a specificity that could reflect the high levels of NAF-1 and BCL-2 in these cells (Bai et al., 2015).

The activation of apoptosis in cells with suppressed NAF-1 expression could account for the decrease in tumor size that is observed in xenograft tumors produced from NAF-1(−) cells (Sohn et al., 2013). Nevertheless, additional factors that could contribute to the reduced size of NAF-1(−) tumors were identified by our metabolomics and transcriptomics analyses. These include the activation of cell cycle arrest genes and the increased AMP:ATP and GMP:GTP ratios that are likely to have resulted from the decrease in mitochondrial function caused by NAF-1 deficiency. The findings that NAF-1(−) cells have an even higher dependency on glycolysis (Figs 5 and 6) compared to control breast cancer cells suggest that anti-glycolytic cancer therapies might be successfully used in conjunction with therapies targeting NAF-1–BCL-2 interactions. The higher dependency on glycolysis observed in NAF-1(−) cells is also interesting because it points to a key role for NAF-1 in regulating energy metabolism in cancer cells, potentially linking the distribution of Fe ions in these cells with energy metabolism.

The suppression of NAF-1 appears to trigger several different HIF1α-, p53- and Nrf2-mediated transcriptional pathways (Fig. 7; Fig. S3, Table S4). These pathways could be triggered by the enhanced ROS production that is due to the accumulation of Fe ions in the mitochondria (Figs 3, 4; Fig. S2). Some of the pathways activated by this process could enhance the transcription of genes involved in glycolysis, which support cellular survival. In contrast, the possible activation of AMPK caused by the increase in the AMP:ATP ratio could induce a starvation response that would inactivate mTOR, promoting cell death (Fig. 7B; Fig. S3). In addition to AMPK, other mechanisms could lead to the induction of apoptosis in NAF-1(−) cells, especially those linked to the stabilization of HIF1α (Fig. 7B). Further studies focusing on the HIF1α, p53, Nrf2 and mTOR pathways are of course required to address these questions.

Our studies suggest that NAF-1 is a major player in the metabolic pathways of cancer cells through its effects on cellular distribution of Fe ions, mitochondrial ROS formation, stabilization of HIF1α and induction of apoptosis. Alterations in NAF-1 expression therefore affect major gene networks and metabolic pathways involved in the survival and proliferation of cancer cells. Further investigation of these pathways will help to elucidate the important roles of NAF-1 in the development and survival of cancer cells, and will possibly help in the development of new drug targets for cancer therapy (for example, see Bai et al., 2015).

## MATERIALS AND METHODS

### Animal studies and cell cultures

Animal experiments were performed in compliance with the Hebrew University Authority for biological and biomedical models (approval number NS-13-13911-4). MDA-MB-231 human breast cancer cells (2.5×10^5^−2.5×10^5^) with normal or suppressed levels of NAF-1 expression were injected subcutaneously into athymic nude (FOXN1NU) 5–6-week-old mice. Sixteen mice were used, eight injected with control cells and eight injected with NAF-1-suppressed cells. Mice weight and tumor size were measured throughout the duration of the experiment. Tumor areas were calculated according to the formula width×length. The animals were euthanized one month after the tumor cell injections. MCF-7 and MDA-MB-231 cells with normal or suppressed levels of NAF-1 expression were generated and grown in culture, as described previously [Sohn et al., 2013; Fig. S4A and B; complementation experiments for the NAF-1(−) lines are shown in Fig. S4C].

### Histology and immunohistochemistry

Subcutaneous tumors were recovered from necropsy and measured for weight and size. Half of the tumor was fixed in 10% formalin and half fixed in 2.5% glutaraldehyde and 2% paraformaldehyde in 0.1 M cacodylate buffer (for TEM analysis, see below). The tissue that had been fixed in 10% formalin was embedded in paraffin blocks, sliced (5 μm) and placed on glass slides. Sections were stained with Hematoxylin and Eosin (H&E). The histological examination was performed by a pathologist (E.P.). Antibodies against phosphorylated histone H2A.X (at Ser139) and activated caspase-3 (Cell Signaling, catalog number 9661) were used to determine cellular apoptosis. An antibody against γH2AX, clone JBW301 (Merck Millipore, Darmstadt, Germany) was used to determine cellular senescence. Antigen retrieval was performed in a microwave in EDTA solution (pH 8, Invitrogen). Horseradish peroxidase (HRP)-conjugated secondary antibodies were used with detection reagent N-Histofine simple stain MAX PO (MULTI) (Nichirei Biosciences). 3,3′-Diaminobenzidine (DAB; Lab Vision) was used as a chromogen.

### Electron microscopy

Tumors that had been fixed for TEM analysis were rinsed four times (10 min each) in cacodylate buffer, post fixed and stained with 1% osmium tetroxide, 1.5% potassium ferricyanide in 0.1 M cacodylate buffer for 1 h and processed for TEM, as described previously (Sohn et al., 2013). Quantitative analysis of mitochondrial damage was performed independently by two investigators, who reviewed each enlarged electron microscopy image for the presence of structurally abnormal mitochondria. The number of damaged mitochondria per image was quantified and analyzed by using ANOVA with the aid of the Origin 8.1 program (OriginLab Corp., MA).

### Fluorescent microscopy

Control and NAF-1(−) MCF-7 and MDA-MB-231 cells were cultured in glass-bottomed microscope dishes in order to assess Fe ion influx by visualizing transferrin receptors and mitochondrial ROS formation with an epi-fluorescent microscope, aided by a confocal (quality equivalent) opti-grid device (Nikon TE 2000 microscope equipped with a thermostated stage and a Hamamatsu Orca-Era CCD camera) and driven by the Volocity 4 operating system (Improvision**,** Coventry, UK), which was used for both image data acquisition and analysis (Sohn et al., 2013). Fe ion influx mediated by transferrin receptors was measured by exposing cells loaded with Fe-ion-sensing red fluorescent RPA to transferrin-bound Fe ions (Breuer et al., 2008; Cabantchik, 2014). A series of time point images were taken over a 30 min time period for each type of cells following the exposure to transferrin-bound Fe ions (2 µM). Mitochondrial ROS formation was determined as described previously (Sohn et al., 2013) using mitoSOX Red (Invitrogen, catalog number M36008). Annexin-V staining to detect apoptosis was performed as instructed by the manufacturer for microscopy analysis (Life Technologies, catalog number A35108) using the EVOS Cell Imaging System. For immunofluorescence analysis, cells were washed with PBS, fixed with 4% paraformaldehyde for 20 min and treated with PBS containing 0.1% Triton X-100 (TPBS) before blocking in 1% BSA for 1 h at 37°C. Cells were incubated with a rabbit polyclonal antibody against TfR1 (Invitrogen) for 2 h at 37°C. A negative control (PBS added) was included. A Cy3-conjugated goat anti-rabbit IgG was used as the secondary antibody and was incubated with cells for 1 h at 37°C. The fluorescent images of cells were acquired using a semi confocal microscope (NiKon Microscopy with Optigrid).

### RNA-Seq and metabolomics analysis

RNA was extracted from cells using the Qiagen RNeasy Mini Kit (catalog number 74104). Three biological replicates were obtained each for control and NAF-1-suppressed cells (each biological replicate was obtained from a pool of three different plates and contained over five million cells each). Paired-end Illumina sequencing generated on average ∼20.9 M read pairs per sample, with each sequence read of a length of 101 nucleotides (http://www.biotech.wisc.edu/gcow). Bowtie (Langmead et al., 2009) was used for alignment of paired-end reads onto the human build-37.2 reference genome; Tophat (Trapnell et al., 2009) was used for parsing the alignment to infer the exon–exon splice junctions, and Cufflinks (Trapnell et al., 2010) was used to perform the differential expression analysis of annotated genes. The abundance of a transcript was measured in terms of ‘fragments per kilobase of transcript per million fragments mapped’ (FPKM), normalized for the transcript length and total number of cDNA fragments for a sample replicate. The raw sequence read datasets and expression results have been deposited to the NCBI GEO database repository and can be accessed with the accession number GSE66158.

For metabolomics analysis, cells were grown as described previously (Sohn et al., 2013) and collected by using trypsinization at confluence for metabolite analysis. Five biological replicates (five million cells each) were obtained for control and NAF-1-suppressed samples, and submitted to Metabolon, Inc. for ultra performance liquid chromatography (UPLC) tandem mass spectrometry (MS/MS) and gas chromatography (GC)-MS analysis (DeHaven et al., 2010; Evans et al., 2009). The liquid chromatography (LC)-MS analysis was performed using a Waters ACQUITY UPLC and a Thermo-Finnigan LTQ mass spectrometer. GC-MS samples were analyzed on a Thermo-Finnigan Trace DSQ fast-scanning single-quadrupole mass spectrometer using electron impact ionization. Raw data was extracted, submitted for peak identification and processed for quality control using Metabolon's hardware and software. Compounds were identified by comparison to library entries of purified standards or recurrent unknown entities. Metabolon maintains a library based on authenticated standards that contains the retention time/index (RI), mass to charge ratio (*m/z*) and chromatographic data (including MS/MS spectral data) on all molecules present in the library. Furthermore, biochemical identification is based on three criteria: retention index within a narrow RI window of the proposed identification, nominal mass match to the library ±0.4 amu, and the MS/MS forward and reverse scores between the experimental data and authentic standards. The MS/MS scores are based on a comparison of the ions present in the experimental spectrum to the ions present in the library spectrum. Although there might be similarities between these molecules based on one of these factors, the use of all three data points can be utilized to distinguish and differentiate compounds. This analysis identified 386 compounds of known identity. Following normalization to Bradford protein concentration, log transformation and imputation of missing values, if any, with the minimum observed value for each compound, ANOVA contrasts were used to identify compounds that differed significantly between NAF-1(−) and empty-vector-transfected cells. Statistical analyses were performed with the program ‘R’ (http://cran.r-project.org/).

The following resources were used for the transcriptomics and metabolomics data analysis: the KEGG Search and Color Pathway tool (http://www.genome.jp/kegg/); GeneCards, The Human Gene Compendium (www.genecards.org); and GeneALaCart (www.genealacart.genecards.org). These resources were used to identify specific pathways that were altered in cells lacking NAF-1 in order to place the different transcripts and metabolites identified within the context of these pathways, and to generate different metabolic maps and figures.

### Cell viability and growth measurements

Wild-type and NAF-1-suppressed MCF-7 and MDA-MB-231 cells were seeded in 96-well plates in triplicate at a density of 3000 cells/well and incubated in RPMI medium with 2-DG at a concentration of 0.5 mM for MCF-7 cells and 0.1 mM for MDA-MB-231 cells. Alamar-Blue (Invitrogen) was used to determine cell viability. Fluorescence was measured on a plate reader after 1–4 h of incubation at 37°C (excitation, 560 nm; emission, 590 nm). For measurements of cell growth, 1 ml of cells was seeded in 24-well plates at 20,000 cells/well. The number of cells in three wells for each treatment per line was counted using a Moxi Z cell counter and type-S cassettes (ORFLO Technologies, Ketchum, ID). 2-DGlucose was purchased from Sigma (catalog number D6134).

### Protein extraction and western blot analysis

Control and NAF-1(−) MCF-7 and MDA-MB-231 cells were grown to 80% confluence on 60 mm Petri dishes. When pertinent, compound C (10 μM) was added to the growth medium 4 h prior to sampling. Medium was aspirated from cultures, and cells were washed with 1× PBS and immediately treated with 1× Laemmli buffer. After 5 min, the lysed cells were swirled together with a 1-ml tip and transferred to a microcentrifuge tube. Samples were heated at 95°C for 10 min, centrifuged for 10 min at 9300 ***g*** and the supernatants were collected. The Pierce 660 nm Protein Assay (catalog number 1861426), Ionic Detergent Compatibility Reagent (IDCR) (catalog number 22663) and Pierce 660 nm Protein Assay Kit were used for protein quantification. Western blotting was performed as described previously (Sohn et al., 2013) using the indicated antibodies against the following proteins: BCL-2 (clone C21; catalog number sc-783, Santa Cruz Biotechnology), BNIP3 (catalog number 13795), p21 Waf1/Cip1 (clone 12D1; catalog number 2947), phosphorylated pS6 (phosphorylated at Ser235 and Ser236) (catalog number 2211), phosphorylated 4E-BP1 (phosphorylated at Thr37 and Thr46) (catalog number 9459), cleaved caspase-3 (cleaved at Asp175) (catalog number 9661), cleaved caspase-7 (cleaved at Asp198) (catalog number 9491), anti-rabbit IgG conjugated to HRP (catalog number 7074). Unless indicated otherwise, all antibodies were obtained from Cell Signaling Technology. Caspase-3 activity was measured using a caspase-3 colorimetric activity assay kit (Chemicon), as per the manufacturer's instructions.

### Statistical analysis

The statistical significance of the fold-change in transcript steady-state levels between two different conditions was assessed for RNA-Seq analysis based on a negative binomial model that had been estimated from the data (Trapnell et al., 2010). The fold-change in the transcription of genes with multiple isoforms was assessed by summing up the FPKMs for all isoforms of a gene and then measuring the difference in this under the two conditions (Trapnell et al., 2010). The statistical significance test for metabolomics analysis was performed using ANOVA (Suzuki et al., 2013). The statistical significance test for protein expression, analysis of TEM images and quantitative PCR were performed by using a one-tailed Student's *t*-test, as previously described (Sohn et al., 2013). Results are presented as mean±s.d. (*P<0.05; **P<0.01; ***P<0.001).
